# Spinal Solitary Plasmacytoma With Minimal Marrow Involvement Presenting With Epidural Spinal Cord Compression

**DOI:** 10.7759/cureus.52460

**Published:** 2024-01-17

**Authors:** Rami Al-Handola, Upasana Banerjee, Yasaman Navari, Sarah Ayad, Huda Marcus

**Affiliations:** 1 Internal Medicine, Hurley Medical Center, Michigan State University/College of Human Medicine, Flint, USA

**Keywords:** extramedullary plasmacytoma (emp), plasmacytoma treatment, solitary bone plasmacytoma, plasma cell dyscrasia, plasma cell neoplasms, spinal solitary plasmacytoma

## Abstract

Solitary plasmacytoma (SPC) is a rare type of plasma cell dyscrasia characterized by the proliferation of neoplastic monoclonal plasma cells. It can involve bone or soft tissue without signs of systemic disease. The solitary bone plasmacytoma typically involves the axial skeleton, most commonly the vertebrae. This article presents a 58-year-old male with a history of Parkinson's disease, hypertension, and cervical spine degenerative joint disease. He arrived at the emergency department with severe thoracic and lumbar back pain, accompanied by numbness and weakness in both legs, which worsened with movement and deep breathing. Magnetic resonance imaging (MRI) findings revealed a sizable mass in the T11 vertebra, leading to thoracic spinal cord compression. Treatment included high-dose dexamethasone, and surgical intervention was undertaken. Subsequent pathology confirmed plasma cell dyscrasia. Radiotherapy and chemotherapy (lenalidomide and dexamethasone) were administered, resulting in no recurrence or new masses after two years. Solitary plasmacytoma is a rare disease with limited clinical trials due to the inability to accrue larger cohorts. Prompt diagnosis and staging of plasmacytomas, involving robust histopathological and radiographic methods, are needed to prevent further complications and possible progression to multiple myeloma. Radiation therapy is the primary treatment, with some studies showing the benefits of lenalidomide and dexamethasone. Further studies are needed to improve treatment options for these patients. This case report adds to the current literature the importance of a multidisciplinary approach to the treatment of SPC.

## Introduction

Solitary plasmacytoma (SPC) is a rare type of plasma cell dyscrasia characterized by the localized proliferation of neoplastic monoclonal plasma cells without systemic plasma cell involvement [1-2]. It can either involve bone or soft tissue without signs of systemic disease [1-2]. Solitary bone plasmacytoma typically involves the axial skeleton, most commonly the vertebrae [1-2]. SPC does not exhibit signs of multiple myeloma (MM), such as CRAB symptoms, including renal insufficiency, hypercalcemia, or anemia, with normal bone marrow morphology or low (<10%) clonal plasma cell infiltration indicating minimal marrow involvement [3]. Diagnosis requires biopsying the suspected lesion with extensive laboratory and imaging studies, adhering to strict criteria. We present a case of spinal solitary plasmacytoma with minimal marrow involvement presenting as epidural spinal cord compression. This case was previously presented as a poster presentation at the Michigan Chapter of the American College of Physicians Annual Scientific Meeting in Bellaire, Michigan, on October 20, 2023.

## Case presentation

**Figure 1:**
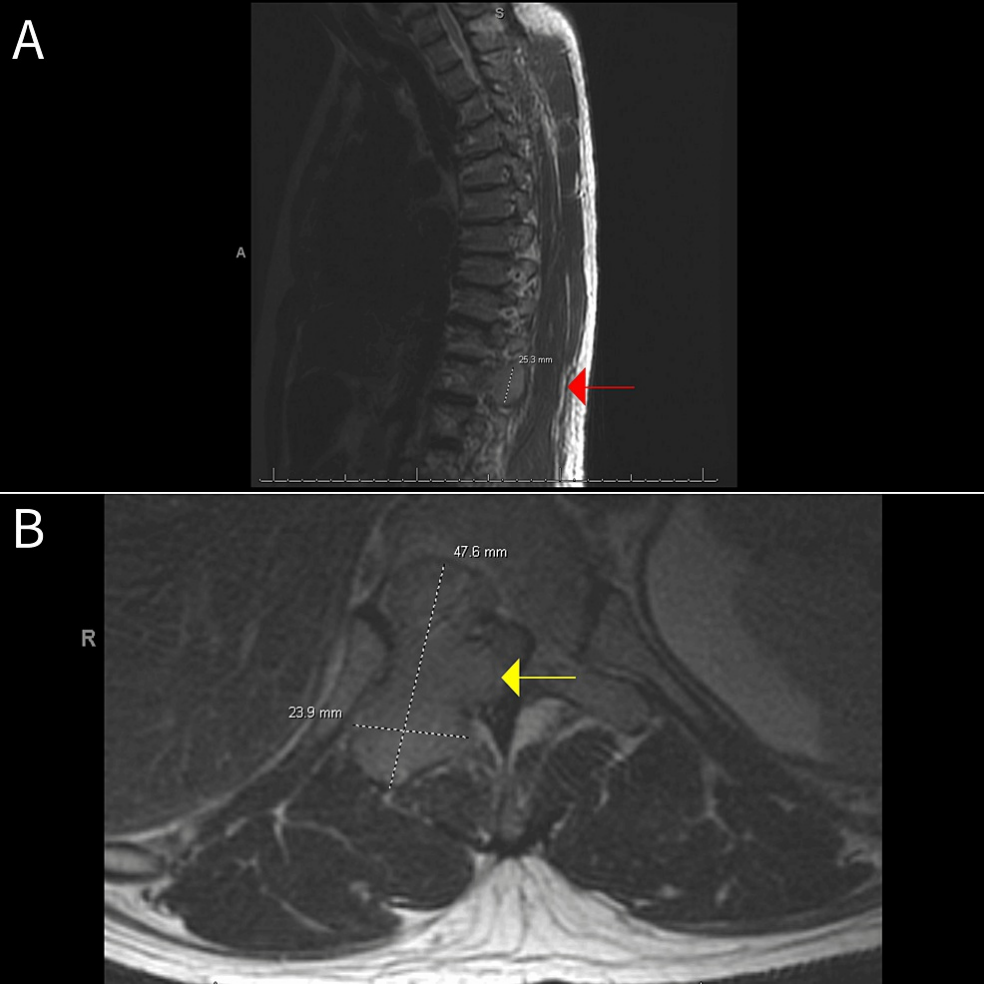
The presence of a 4.8 x 2.4 x 2.5 cm lytic expansile and heterogeneously enhancing mass with abnormal bone marrow signal intensity involving the posterior right T11 vertebral body extending into the right posterior element (red arrow in A) with abnormal signal extending into the dorsal aspect of the spinal canal resulting in a mass effect on the thoracic spinal cord (yellow arrow in B).

A 58-year-old male with a past medical history of Parkinson's disease with a deep brain stimulator, hypertension, and cervical spine degenerative joint disease status post laminectomy presented to the emergency department with worsening back pain of four weeks' duration, affecting the thoracic and lumbar spine. The pain was associated with numbness extending to the thighs and weakness of the bilateral lower extremities and has been aggravated by ambulation, bending, and deep breathing, with no alleviating factors reported. He denied fever, pelvic or leg pain, bowel or bladder control loss, and perineal numbness or tingling. There is no history of falls, injuries, cancer, steroid use, or vascular diseases. Laboratory results showed elevated C-reactive protein and erythrocyte sedimentation rate with the absence of hypercalcemia, anemia, and renal dysfunction. Magnetic resonance imaging (MRI) of the spine with and without contrast revealed a large heterogeneous enhancing mass with a lytic expansile lesion in the T11 vertebra, causing stenosis and compression of the thoracic spinal cord (Figure 1). The patient was loaded with dexamethasone 10 mg IV push followed by dexamethasone 4 mg every six hours.

Surgical intervention involving pedicle screw fusion at T8-L2, T11 laminectomy, and an open partial resection with a biopsy of the T11 mass was performed for both diagnostic and therapeutic purposes. Immunohistochemistry staining was positive for CD10. Flow cytometry revealed plasma cells with CD38 expression, showing monoclonality with Lambda light chain restriction. More than 80% of plasma cells were positive for CD45. Bone marrow biopsy revealed increased marrow plasma cells (5%), with a clonal population by flow cytometry, consistent with plasma cell dyscrasia. Immunophenotyping by flow cytometry revealed a clonal plasma cell population expressing CD56 and monotypic lambda light chain. The plasma cells were negative for the kappa light chain. Chromosome analysis of bone marrow showed no consistent numerical or structural chromosome abnormalities. Myeloma profile interphase fluorescence in situ hybridization (FISH) analysis was abnormal with loss of Neurofibromatosis type 1 (NF1)/17q and gains of chromosomes 5, 9, 11, and 15. A spike of 0.9 grams, followed by a repeat value of 1.4 grams, was noted on serum protein electrophoresis (SPEP), with lambda and IgG elevated. Serum immunofixation detected IgG lambda monoclonal immunoglobulin. Serum-free light chains showed elevated free lambda chains and a low kappa/lambda ratio. No other lesions of bony involvement were detected in the computed tomography (CT) scan of the chest, abdomen, pelvis, or positron emission tomography (PET) scan. The patient was started on lenalidomide 15 mg orally daily with a three-week-on and one-week-off pattern, as well as dexamethasone 20 mg weekly. Concurrently, radiotherapy (RT) was started at the site of the decompressive laminectomy; he was planned for 23 fractions with a total of 46 Gray (Gy). The two-year follow-up did not show any recurrent or new masses.

## Discussion

SPC is a rare disease considered part of plasma cell neoplasms. Plasma cell neoplasms refer to the neoplastic proliferation of a single clone of plasma cells. These disorders can present as either a single lesion, an SPC lesion, or multiple lesions, such as MM. SPC commonly occurs in the bone (osseous plasmacytoma) but can also occur outside the bones, as in soft tissue, and is called an extramedullary plasmacytoma [1]. In the bone, it is more common in active hematopoietic bones such as the axial skeleton of the vertebra and skull than in the appendicular skeleton. SPC accounts for approximately 5% of plasma cell disorders. It is more common in males and the Black race [1]. The median age of diagnosis is between 55 and 65 years [1,2], with an incidence of 0.15 cases/100,000 person/year [2]. Presentation usually occurs with bone pain or a pathological fracture of the affected bone. Vertebral involvement can carry the risk of neurological impact, such as in our patient.

Diagnosis of SPC requires a clonal plasma cell solitary bone or soft tissue tumor proved by biopsy, lack of other lytic or extramedullary lesions, bone marrow biopsy with no or clonal plasma, and the absence of the classical CRAB manifestations including hypercalcemia, renal insufficiency, anemia, or bone lesion that can be attributed to a lymphoplasma cell proliferative disorder [3]. On the other hand, solitary plasmacytoma with minimal marrow involvement has similar diagnostic criteria, with the exception that bone marrow can contain clonal plasma cells less than 10% [3]. About half of SPC patients will progress to MM within two to three years [4]. SPC with minimal marrow involvement carries more recurrence risks and/or progression to multiple myeloma.

RT is the primary treatment for SPC [5]. However, even with adequate RT, more than half of SPC patients transform into MM. Therefore, several researchers have combined RT with systemic therapy to enhance the prognosis of SPC. The use of chemotherapy remains controversial, but some studies have shown benefits of lenalidomide and dexamethasone in SPC treatment, such as improving local control, multiple myeloma-free survival, and progression-free survival rates [6-8], both medications were used in our patient.

The role of surgery in SPC is unclear; however, in spinal SPC, surgery can play a role in cord decompression and tumor resection, reducing pain and symptoms and improving quality of life [9-10], but these patients should also receive the main treatment, which is radiation, for complete eradication. The exact dose of RT is not known; moderate-dose radiation is the usually preferred choice, according to the National Comprehensive Cancer Network panel recommendations, 40-50 Gy in 1.8-2.0 Gy/fraction to the involved field. The overall survival rate is about 74% at five years and 45% at ten years [11-13]. Patients should be followed up for surveillance every three to six months with blood, urine, and imaging studies to verify the reappearance or appearance of M-protein and confirm that local control was obtained after RT [13].

## Conclusions

SPC is a rare disease that affects only a small number of patients. When SPC involves the bone marrow, it becomes even rarer. Due to its rarity, there have been limited clinical trials on the disease. RT is the primary treatment, but it is now being combined with systemic therapy, although this approach is controversial due to insufficient data, with aims to improve the prognosis of SPC. However, the progression to MM remains high. Further studies are required to improve the available treatment options and enhance the chances of survival, as prolonging survival and maintaining the quality of life for patients is crucial. Therefore, it is vital to continue exploring and documenting every possible experience in the literature to collect more information and experiences about SPC and develop better ways to manage it.
